# Phylodynamic analysis provides insights into genotype displacement of duck hepatitis A virus in China

**DOI:** 10.4142/jvs.25210

**Published:** 2026-08-03

**Authors:** Chenyi Xie, Baoyan Wang, Ying Wang, Guangrong Zhao, Yang Liu, Jianlong Zhang, Xin Yu, Linlin Jiang, Jiayu Yu, Jiuyu Zhan, Hongwei Zhu, Xingxiao Zhang

**Affiliations:** 1 School of Life Sciences, Ludong University, Yantai 264025, China.; 2 Yantai Key Laboratory of Animal Pathogenetic Microbiology and Immunology, Yantai 264025, China.

**Keywords:** Duck hepatitis A virus, *vp1*, genotype displacement, phylodynamic analysis, molecular epidemiology

## Abstract

**Importance:**

Duck viral hepatitis, caused by the duck hepatitis A virus (DHAV), poses a significant threat to the global duck industry, particularly in China.

**Objective:**

This study examined the evolutionary mechanisms driving the genotype displacement of DHAV-1 by DHAV-3 in China using phylodynamic analysis.

**Methods:**

The *vp1* genes of 9 DHAV-1 and 17 DHAV-3 strains were sequenced and combined with 82 DHAV-1 and 86 DHAV-3 sequences from GenBank. Bayesian phylogenetic, demographic, and phylogeographic analyses were performed using BEAST v1.10.

**Results:**

The substitution rate for DHAV-1 *vp1* was 4.89 × 10^−4^ substitutions/site/year (95% highest posterior density [HPD]: 3.072 × 10^−4^ to 6.83 × 10^−4^), and for DHAV-3 was 2.125 × 10^−4^ substitutions/site/year (95% HPD: 1.686 × 10^−4^ to 2.567 × 10^−4^). The time to the most recent common ancestor for DHAV-1 was estimated at ~1911 and for DHAV-3 at ~1992. The demographic trends showed a decline in DHAV-1 after 2003 and a resurgence of DHAV-3 in ~2010. Phylogeography identified China as the hub for DHAV-3 spread to South Korea, Vietnam, and Egypt.

**Conclusions and Relevance:**

DHAV-3 has a higher evolutionary rate, and adaptive mutations drive its dominance, informing vaccine design and control strategies.

## INTRODUCTION

Duck viral hepatitis (DVH), caused by the duck hepatitis A virus (DHAV), is a highly lethal enzootic disease primarily affecting ducklings. Characterized by rapid onset, mortality rates often exceeding 95% in young ducks, and extensive hepatic necrosis [1], DVH poses a significant threat to the global duck industry. This impact is particularly pronounced in China, the world’s leading producer of domestic ducks [2,3,4]. Consequently, DVH necessitates ongoing surveillance and robust control strategies to mitigate its economic and biological consequences.

Considering the severe impact of DVH, understanding the virological properties of DHAV is critical for effective disease management. DHAV, a member of the family *Picornaviridae* and the genus *Avihepatovirus*, has a linear, single-stranded positive-sense RNA genome approximately 7.8 kb in size that consists of a large open-reading frame encoding a polyprotein and untranslated regions [5,6]. The polyprotein is processed into capsid proteins VP0, VP3, and VP1 and non-structural proteins 2A1, 2A2, 2A3, 2B, 2C, 3A, 3B, 3C, and 3D. VP1 [7,8], the most accessible surface capsid protein of picornaviruses, is considered the major antigenic determinant and, to some extent, plays roles in viral pathogenesis and virulence [9]. Previous studies have confirmed that the VP1 protein of DHAV is involved in receptor binding and contains conserved B-cell epitopes [5,10]. Genetic characterization of the *vp1* gene has been used extensively for DHAV genotyping and molecular epidemiological studies [11].

The evolutionary dynamics of DHAV genotypes are central to understanding DVH epidemiology [12]. Viral genotype displacement involves replacing a dominant genotype with a novel one in a geographic region. DHAV is classified into three genotypes: classical DHAV-1 [13], Taiwan-exclusive DHAV-2, and DHAV-3 from South Korea and mainland China. Historically, DHAV-1 dominated global DVH outbreaks, but recent Chinese surveillance has shown DHAV-3 emerging as the predominant strain, displacing DHAV-1. This shift raises questions about mechanisms, such as antigenic variation, recombination, or host immune adaptation, that underlie the fitness of DHAV-3.

To answer these questions, advanced analytical approaches are needed to uncover the factors shaping the Genotype displacement of DHAV in China. Phylodynamic analysis [12,13], which integrates phylogenetics with epidemiological dynamics, has been instrumental in deciphering the spatiotemporal spread and the evolutionary pressures shaping viruses, e.g., influenza virus and coronaviruses. Despite its potential, this approach remains underutilized in studying DHAV, a virus of high genetic diversity and aquacultural significance. Current gaps include understanding how DHAV-3 achieved dominance over DHAV-1 in China and identifying selective drivers, such as vaccination practices or host-pathogen coevolution, that may influence genotype competition.

The evolutionary and epidemiological dynamics of the virus based on 102 genomes of DHAV strains sampled from China were used to understand the molecular epidemiology of DHAV-1 and DHAV-3 strains circulating and genotype displacement in China. The mechanisms driving genotype displacement were determined by reconstructing viral evolutionary trajectories, estimating population dynamics, and mapping adaptive mutations. The results showed that the expansion of DHAV-3 correlates with accelerated evolutionary rates and key antigenic site mutations. These insights enhance the understanding of DHAV evolution and inform targeted vaccine development and surveillance strategies to mitigate its impact on global duck aquaculture.

## METHODS

### Sample collection and virus isolation

From 2006 to 2019, 102 liver samples were collected from domestic ducklings with typical clinical signs of viral hepatitis from farms in Anhui, Guangxi, Hebei, Jiangsu, Jiangxi, Shandong, and Sichuan Provinces in China. For virus isolation, the livers were homogenized in phosphate-buffered saline, then clarified by centrifugation at 7,500 × *g* for 20 min. The supernatants were filtered using a syringe filter unit (0.22 μm pore-size) and were propagated in the allantoic cavities of 10-day-old duck embryos for 3–4 days. Allantoic fluid was then collected for the subsequent RNA isolation or stored at −80°C until used.

The total RNA was prepared from DHAV isolates (first passage in duck embryos) or liver samples with TRIzol Reagent (Ambion, USA) according to the manufacturer’s instructions. The complementary DNA was synthesized using the SuperScript IV Reverse Transcriptase (Invitrogen, Lithuania) in a 20 μL reverse transcription reaction. Briefly, 50 μM random hexamers, 5 μg of total RNA, and 10 μM dNTP mix were heated at 65°C for 5 min and incubated on ice to anneal the primers to the template RNA. RNaseOUT Ribonuclease Inhibitor (40 U), DTT (100 μM), and SuperScript IV Reverse Transcriptase (200 U) were then added to the reaction mixture and incubated at 23°C for 10 min, followed by a second incubation at 55°C for 10 min and heat inactivation.

Before *vp1* amplification, the genotypes of these isolates were first determined based on the sequence alignment matches queried in the BLASTN database. Genotype-specific nested polymerase chain reaction (PCR) was then used to amplify the DHAV-1 or DHAV-3 *vp1* coding region with PrimeSTAR HS DNA Polymerase (Takara, China) and a set of degenerate PCR primers (Supplementary Table 1). PCR amplicons were purified and cloned into the pCR-Blunt vector (Invitrogen, USA). The 26 sequences determined were deposited in GenBank under the accession numbers MN912702–MN912703, MN335931–MN335932, and MK903049–MK903070.

### Genotype statistics

All available DHAV sequences were retrieved from the Entrez Molecular Sequence Database System at the National Center for Biotechnology Information website using “duck hepatitis A virus” or “Avihepatovirus A” as keywords to obtain the genotype distribution information for DHAV field isolates circulating within Chinese domestic duck flocks. The vaccine strain, serial-passaged isolates, and duplicated isolates were excluded. The genotype information of all Chinese isolates was retrieved and analyzed statistically.

### Datasets collation, recombination detection, and temporal signal

For DHAV *vp1* gene evolutionary reconstruction, 9 full-length DHAV-1 *vp1* gene (714 nucleotides, nt) were determined in this study, and the other 82 GenBank records constituted dataset 1. Dataset 2 consisted of 11 sequenced 720 nt DHAV-3 *vp*1 and 92 publicly available sequences. Dataset 3 consisted of a truncated *vp1* fragment (669 nt) of 173 DHAV-3 isolates with specific time and geographic information. Dataset 4 comprised 195 DHAV-1 and DHAV-3 *vp1* codons used for selection analysis (Supplementary Table 2). Sequences derived from serial passages of viruses or vaccine strains were excluded from these datasets. Identical sequences were also collated, leaving unique sequences in datasets in case of viral clones. RDP4 software was used to detect recombination or genomic reassortment signals in both datasets [14]. The datasets were then assessed using TempEst v1.5.3 [15] for the temporal signal by estimating the root-to-tip regression. The results suggested that both data sets exhibited a positive correlation between genetic divergence and sampling time and were suitable for phylogenetic molecular clock analysis, but DHAV-3 (dataset 2) showed a stronger association between genetic distances and sampling dates than DHAV-1 (dataset 1) with correlation coefficients of 0.79 and 0.17 respectively. Supplementary Table 1 lists the locations, accession numbers and other details of these isolates.

### Bayesian evolutionary analysis

Maximum clade credibility (MCC) trees for datasets 1 and 2 were reconstructed using the Markov chain Monte Carlo (MCMC) sampling method implemented in the BEAST package v1.10 [16]. The substitution models for datasets 1 and 2 were HKY+F+Γ4 and TN+F+Γ4, as inferred by the Bayesian information criterion values estimated by ModelFinder [17]. For the most appropriate clock and demographic model combination, a marginal likelihood estimation was performed using path sampling and stepping-stone sampling for comparing strict and relaxed (uncorrelated log-normal distribution) clock models and four coalescent models (constant size, Bayesian skyline plot [BSP], exponential growth, and Gaussian Markov Random Field [GMRF] Bayesian skyride) [18,19,20]. The results suggested that the strict clock model and the Bayesian skyline coalescent model were the best model combination for dataset 1, while the uncorrelated relaxed log-normal clock model and the GMRF Bayesian skyride coalescent model fitted dataset 2 best (Supplementary Table 3). Under these models, the MCMC chains were run for 100,000,000 iterations and assessed for good mixing using an adequate effective sample size in Tracer v1.6, ignoring 10% of the chains as burn-in. Evolutionary histories were summarized in annotated MCC trees using TreeAnotator v 1.8.0, which could be visualized in FigTree v 1.4.4. Posterior probability values were used to evaluate the statistical support of each node on the tree.

### Demographic history

For a demographic history estimation of DHAV-1 and -3 over time, BSP and GMRF Bayesian Skyride were implemented in Tracer software using the log files from the MCMC sampling procedures for datasets 1 and 2, as described above. The changes in population size were visualized by plotting the changes in genetic diversity as a function of time.

### Phylogeny-geography correlation

The association between phylogeny and geography of DHAV-3 was investigated using the Bayesian Tip Significance testing (BaTS) method [21]. Using the phylogeny from dataset 3 generated by the BEAST package, the geographical correlation was analyzed by calculating the statistical significance of the null hypothesis that the traits are randomly associated with phylogeny tips. The parsimony score (PS), association index (AI), and maximum monophyletic clade (MC) size were reported, and *p* values < 0.05 indicated the significance of phylogeny-trait associations (Supplementary Table 4).

### Discrete phylogeographic analysis

For the spatial dispersal analysis of DHAV-3 among China, South Korea, Vietnam, and Egypt, discrete phylogeographic analysis was performed using a Bayesian MCMC approach in the BEAST package v1.10 [16]. Briefly, a discrete diffusion model was applied to a symmetric substitution model using a standard continuous-time Markov chain (CTMC). The MCMC was also used to infer a social network using the Bayesian Stochastic Search Variable Selection (BSSVS) procedure, which identified the most parsimonious description of the phylogenetic diffusion process (Supplementary Table 5) [22]. After adequate chain mixing, an MCC tree was summarized, visualized, and annotated, as described above. Spatial dynamics supported by the Bayes factor (BF) test were analyzed using SPREAD v1.0.6 [23].

### Selective pressure

To compare the selective pressure posed on the *vp1* codons of DHAV-1 and DHAV-3 during the evolutionary process. EasyCodeML, a Java-based version of CODEML in the PAML package, was used to calculate the ratio (ω) of dN (the non-synonymous rate)/dS (the synonymous rate) on the selected branch and positively selected codons using branch mode, branch-site mode, site mode, and clade mode [24,25]. The fixed effects likelihood (FELE) was chosen and run to estimate the (dN-dS) site-by-site selection at every codon for both datasets. Codons showing evidence of positive or negative selection were reported using the default significance levels. The Mixed Effects Model of Evolution (MEME) module was also used to detect the individual sites subject to episodic diversifying selection (Supplementary Table 6) [26].

## RESULTS

### Evolutionary history of China-related DHAV

The evolutionary history of the China-related DHAVs was inferred in 2 time-scaled MCC trees. The mean nucleotide substitution rate/site/year of the full-length DHAV-1 *vp1* gene was estimated to be 4.890 × 10^−4^ with the 95% HPD ranging from 3.072 × 10^−4^ to 6.830 × 10^−4^ (Table 1). Under these substitution rates, the time to the most recent common ancestor (tMRCA) of the China-related DHAV-1 *vp1* gene was estimated to have circulated around 1911, with a 95% highest posterior density (HPD) interval ranging from 1864 to 1944 (Table 1). Among the China-related clade, two major clusters (virus groups) were identified: clade 1 and clade 2. In addition, 7 subgroup clusters were further identified within these groups. The mean nucleotide substitution rate/site/year of the full-length DHAV-3 *vp1* gene was predicted to be 2.125 × 10^−4^, with the 95% HPD distributed from 1.686 × 10^−4^ to 2.567 × 10^−4^. The tMRCA of the China-related DHAV-3 *vp1* gene was estimated to have circulated around 1992, with a 95% HPD interval ranging from 1989 to 1996 (Table 1). Among the China-related clades, 3 major clusters were identified: clades 1, 2, and 3. Seven subgroup clusters were identified within these groups (Fig. 1A and B).

**Table 1:** Evolutionary rate comparison between DHAV-1 and DHAV-3

Virus/genotype	tMRCA (year)		Substitution rate (substitutions/site/year)	
	Mean	95% HPD	Mean	95% HPD
DHAV-1-*vp1*	1911	1864 to 1944	4.89E-04	3.072E-04 to 6.83E-04
DHAV-3-*vp1*	1992	1989 to 1996	2.12E-04	1.686E-04 to 2.567E-04

DHAV, duck hepatitis A virus; tMRCA, time to the most recent common ancestor; HPD, highest posterior density.

**Fig. 1:**
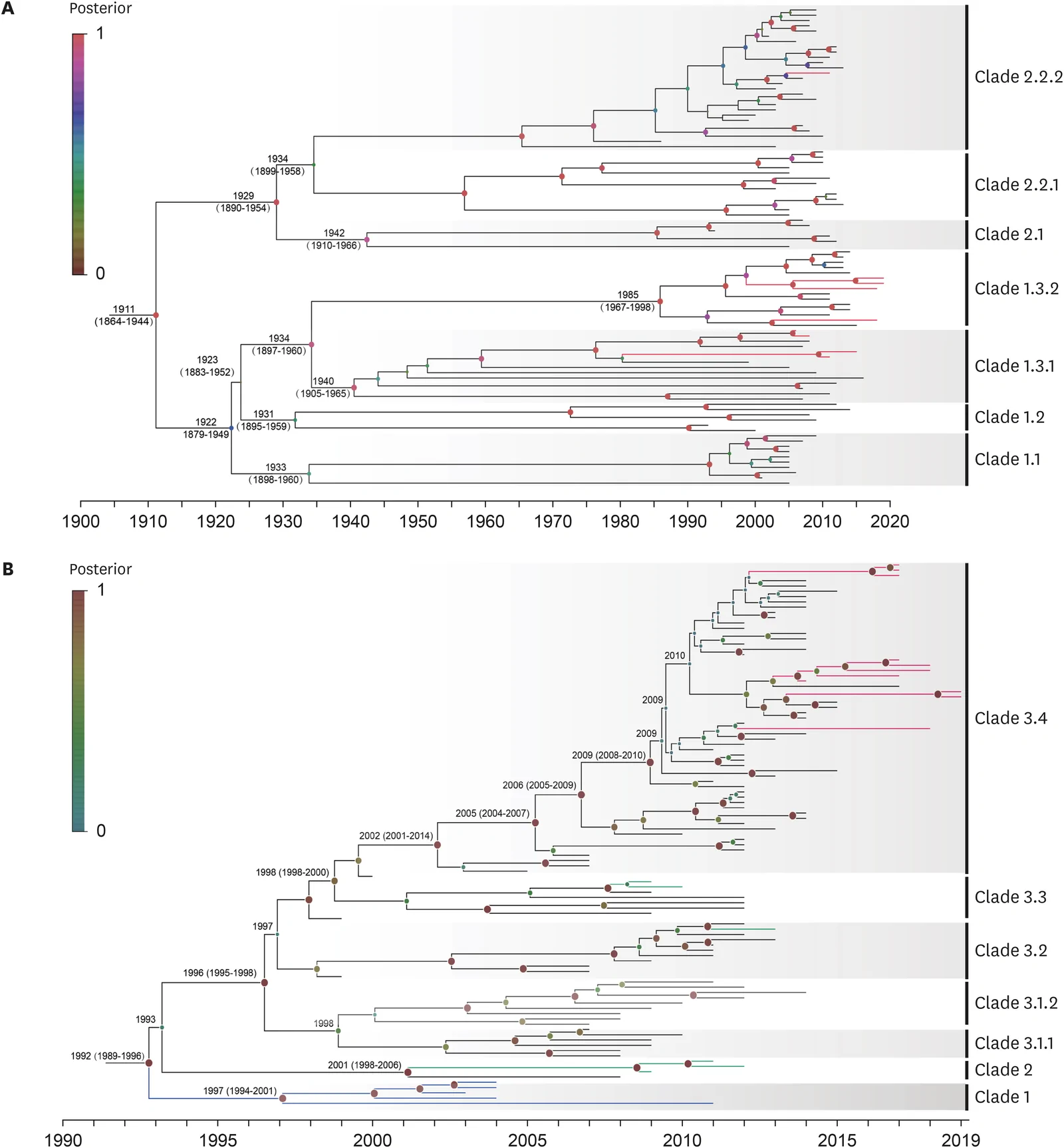
Time-scaled maximum clade credibility phylogeny of China-related duck hepatitis a virus using 194 complete coding sequences of the *vp1* gene from datasets 1 and 2. The horizontal time scale shows the estimated divergence year, and the vertical bars define the clustered groups within the lineage. Divergence dates for the major branches together with their 95% highest posterior density ranges are indicated above and below the tree nodes. Posterior probabilities are shown as the sizes of the circles and node colors according to the legend on the left. Selected values in the major branches are also shown as numbers. The red line indicates the strains that were collected for the present experiment.

The DHAV-1 strains isolated in this study were predominantly clustered within clades 1.3.1 and 1.3.2, with a sporadic distribution in clade 2.2.2 (Fig. 1A). In contrast, the DHAV-3 strains were exclusively localized to clade 3.4 within the broader clade 3 (Fig. 1B). The DHAV-1 strains evolved much earlier than the DHAV-3 strain.

### Population demography of China-related DHAVs

The demographic history of China-related lineages was investigated and presented as BSPs, which help estimate effective population size over time. Population dynamic analyses revealed distinct temporal trends in the prevalence of DHAV-1 and DHAV-3 genotypes. For DHAV-1, a gradual decline in viral abundance was observed around 2003, followed by stabilization prior to 2010 (Fig. 2A). In contrast, DHAV-3 followed a similar gradual decline around 2007, succeeded by a moderate resurgence near 2010 and a subsequent stabilization with a slight downward trajectory by 2012 (Fig. 2B).

**Fig. 2:**
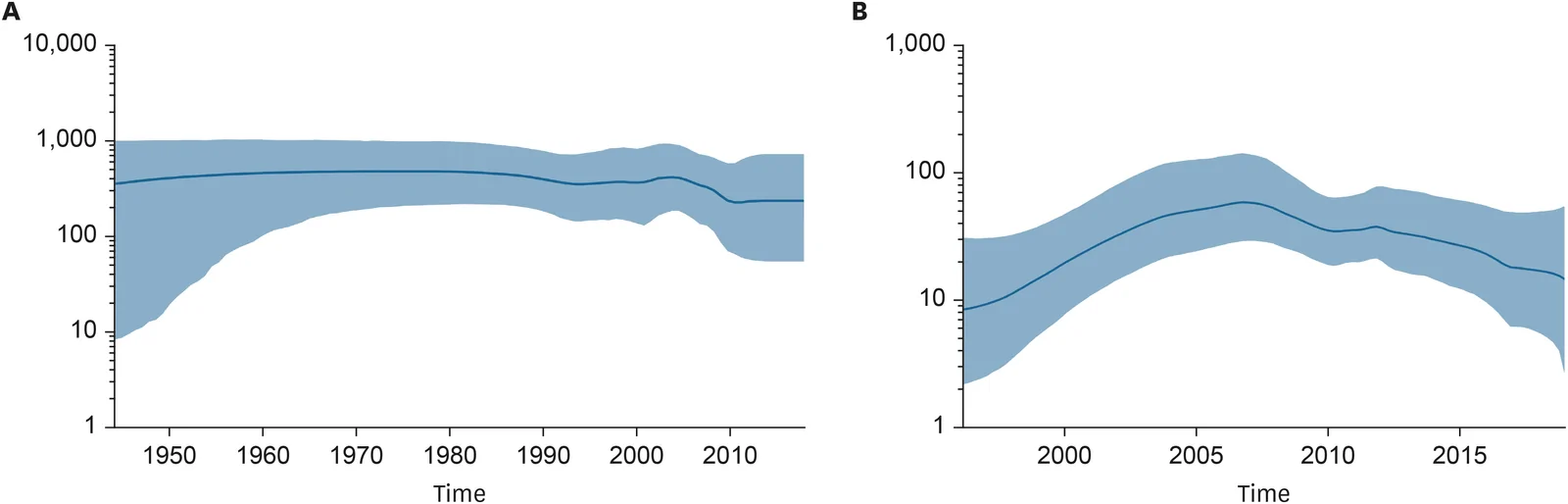
Demographic history of DHAV-1 (A) and DHAV-3 (B) evaluated by BSPs. Dynamics of the *vp1* gene genetic diversity were estimated by the BSP and Gaussian Markov Random Field Bayesian skyride. The analysis was performed using the trees log file generated by the Markov chain Monte Carlo analysis as described. Dynamics of the mean genetic diversity are shown as a black line, and the estimated 95% highest posterior density intervals are presented as the solid blue areas. The height and width of the plot are proportional to the effective population sizes and time scales. DHAV, duck hepatitis a virus; BSP, Bayesian skyline plot.

### Selection pressure through the evolutionary process

Codon-based maximum-likelihood analysis was performed using site models M0, M1a, M2a, M3, M7, M8, and M8a to investigate the selection pressure acting on the *vp1* genes of DHAV-1 and DHAV-3. For DHAV-1 *vp1*, the positively selected sites identified under M8 were S49, L181, Q183, G184, V187, N193, and Y219, suggesting that these residues may be subjected to host-driven evolutionary pressure. For DHAV-3 *vp1*, positively selected sites included S49, Q185, L186, N188, I189, N196, and K207, indicating potential functional adaptation in response to host immune pressures (Supplementary Table 5).

The positive selection sites in the *vp1* genes of DHAV-1 and DHAV-3 were assessed using site-specific selection analyses with FUBAR, MEME, FELE, and SLAC methods. For DHAV-1 *vp1*, three sites, S49, Q183, and N193, showed significant evidence of positive selection. In particular, sites Q183 (FUBAR = 0.994, MEME *p* = 0.012, FELE *p* = 0.0197) and N193 (FUBAR = 0.995, MEME *p* = 0.02, FELE *p* = 0.0126) had high posterior probabilities (> 0.95) in FUBAR and significant *p* values in MEME and FELE, providing strong evidence of adaptive evolution at these residues. For DHAV-3 *vp1*, two sites, S49 and I189, exhibited positive selection signals. Among them, 49S (FUBAR = 0.992, MEME *p* = 0.036, FELE *p* = 0.024) and I189 (FUBAR = 0.965, MEME *p* = 0.026, FELE *p* = 0.044) were detected using multiple models, suggesting their potential role in viral adaptation: Geographic Clustering and Transnational Transmission Dynamics in Asia and Egypt. The association of phylogeny and geography of DHAV-3 was calculated using the BaTS method. The observed values of AI and PS indices from the estimated MCC trees for the test were significantly lower than the null values (*p* = 0), indicating the rejection of the null hypothesis of no association between phylogeny and geography (Supplementary Table 4). The correlation between phylogeny and geographic structure suggests that DHAV-3 is mostly clustered by geography. The phylogeny-trait association for location was also calculated using maximum MC size statistics.

The spatial dispersal patterns of DHAV-3 among China, South Korea, Vietnam, and Egypt were elucidated using discrete phylogeographic analysis with a Bayesian MCMC approach implemented in BEAST v1.10. A discrete diffusion model was applied under a symmetric substitution model using a CTMC. BSSVS was used to identify the most parsimonious phylogenetic diffusion process, thereby allowing inference of the most probable viral migration pathways. The MCMC analysis achieved adequate chain mixing, and a MCC tree was summarized and visualized as previously described.

The results revealed significant spatial dispersal events, with strong Bayesian support for viral migration between China and South Korea, and between China and Vietnam. The BF test conducted using SPREAD v1.0.6 confirmed that these transmission routes had substantial statistical support (BF > 10), indicating strong viral movement between these geographic regions. In addition, sporadic viral introductions were observed between China and Egypt, even though these pathways had lower BF support, suggesting less frequent or transient transmission events.

Further analysis of the phylogenetic tree topology suggested that China served as a central hub for DHAV-3 dissemination, with multiple viral strains originating in China and subsequently spreading to South Korea and Vietnam. This finding is consistent with the hypothesis that China plays a dominant role in the regional evolution and circulation of DHAV-3 (Fig. 3). In contrast, viral movements to Egypt were relatively limited, possibly because of geographical barriers or differences in poultry trade patterns.

**Fig. 3:**
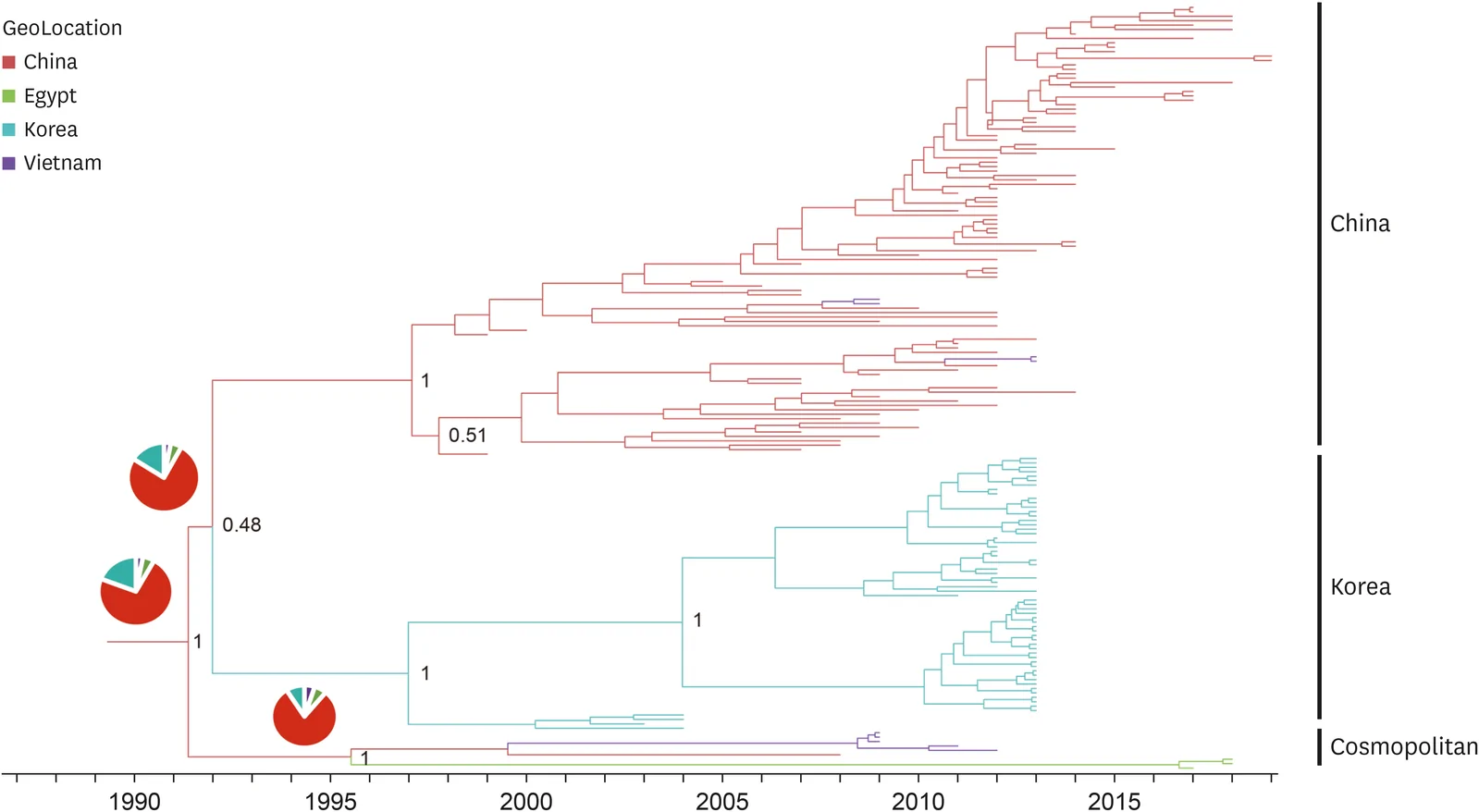
Maximum clade credibility phylogeny of 173 partial *vp1* gene sequences of duck hepatitis a virus-3 presented using discrete phylogeography. The discrete phylogeographic analysis was performed using a Bayesian Markov chain Monte Carlo approach in the BEAST package v1.10. Colors of the branches represent the most likely location states of the descendant nodes: China (red), Egypt (green), Korea (blue), and Vietnam (purple). The major clades are labeled based on their predominant geographic distributions. Pie charts indicate the proportion of geographic origins within key nodes.

### Genotype displacement of DHAV in China

From 1986 to 2011, genotype DHAV-1 dominated as the epidemiologically predominant strain in China. This dominance shifted in 2012 when genotype DHAV-3 began displacing DHAV-1, and it has since maintained its epidemiological supremacy (Fig. 4A). Specifically, DHAV-3 was first detected in China as early as 1999 (Fig. 4B). Since 2013, the detection frequency of duck hepatitis viruses has increased significantly, coinciding with disease outbreaks. During the epidemic period, the detection rate of DHAV-3 markedly outnumbered that of DHAV-1, suggesting that DHAV-3 exhibits enhanced transmissibility compared to DHAV-1.

**Fig. 4:**
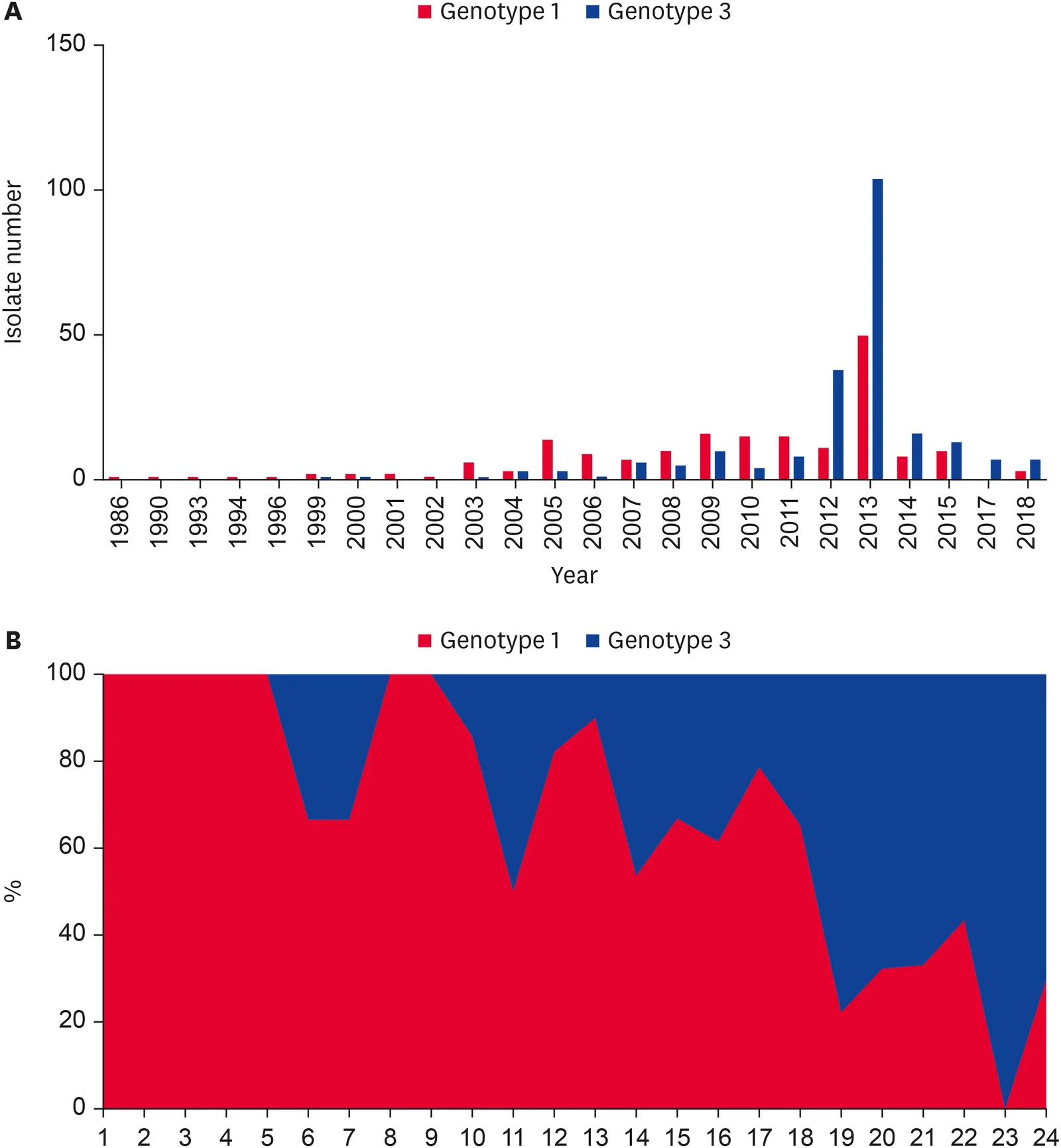
Genotype displacement and epidemiologic dynamics of DHAV in China (1986–2018). The annual number of isolates for each genotype was statistically analyzed and visualized using GraphPad Prism 10. (A) Annual number of DHAV genotype 1 (DHAV-1, green bars) and genotype 3 (DHAV-3, blue bars) isolates. (B) Stacked area chart illustrating the proportional distribution (%) of each genotype over time, highlighting the shift in dominant circulating strains. DHAV, duck hepatitis A virus.

## DISCUSSION

This study revealed a clear genotypic displacement of DHAV in China, with DHAV-3 having supplanted DHAV-1 as the predominant genotype since approximately 2012. This shift coincides with several key evolutionary and epidemiological factors.

Bayesian coalescent analyses suggest that contemporary Chinese DHAV-1 lineages tMRCA around ~1911, whereas DHAV-3 emerged much more recently, circa 1990–1992, within China itself. In particular, the VP1 gene of DHAV-3 evolves at a rate of (2.125 × 10^−4^ substitutions/site/year), approximately twofold faster than DHAV-1 (4.89 × 10^−4^ substitutions/site/year). This elevated evolutionary rate likely enhances the capacity of DHAV-3 for rapid genetic diversification and adaptation, conferring a competitive advantage over DHAV-1.

Population demographic reconstruction using BSPs and annual isolate counts showed that the prevalence of DHAV-1 began declining around 2003, stabilizing at low levels after 2010 [27]. In contrast, DHAV-3 experienced a temporary dip around 2007, followed by a resurgence and eventual dominance from 2012 onward. The widespread deployment of live-attenuated DHAV-1 vaccines (CH60 and A66 strains) since 2013 probably exerted strong selective pressure on circulating field viruses. Limited cross-protection against the antigenically distinct DHAV-3 may have facilitated immune escape and the subsequent rise of this genotype, a pattern repeatedly observed when monovalent vaccines are used against antigenically diverse viral populations [28].

Evidence of adaptive evolution in the VP1 protein, particularly at codons under episodic or positive selection (e.g., position 49 in both genotypes, and 183, 193 in DHAV-1; 189 in DHAV-3), further supports the hypothesis that DHAV-3 has evolved enhanced fitness and immune-evasion capabilities. These findings align with observations in other picornaviruses, where antigenic drift in capsid proteins drives lineage turnover under vaccine-induced immunity [29].

Phylogeographic reconstruction confirms China as the epicenter of DHAV-3 diversity and dissemination. Strongly supported migration links exist between mainland China, South Korea, and Vietnam (BF > 10), consistent with intensive regional poultry trade. In contrast, introductions into Egypt appear sporadic and less sustained, highlighting the role of trade networks in structuring viral spread across East/Southeast Asia and, to a lesser extent, North Africa.

In conclusion, the displacement of DHAV-1 by DHAV-3 in China reflects a combination of a higher evolutionary rate, adaptive mutations in the VP1 protein, and vaccine-mediated selection pressure. These insights underscore the need for updated, bivalent, or broadly protective vaccines and emphasize the importance of coordinated regional surveillance to mitigate further international spread of emergent DHAV-3 lineages.

## SUPPLEMENTARY MATERIALS

Supplementary Table 1 Oligonucleotide primers used in this study

Supplementary Table 2 Sequences included in the datasets in this study

Supplementary Table 3 Marginal likelihood estimation of the DHAV-1 and DHAV-3 VP1 coding sequence datasets using path sampling/stepping-stone sampling

Supplementary Table 4 Estimated geographic structure of duck hepatitis A virus-3 based on the 173 truncated *vp1* fragment (669 nt)

Supplementary Table 5 Migrations well supported by Bayes factor tests^a^

Supplementary Table 6 Positively selected sites analysis of the DHAV-1 VP1 and DHAV-3 VP1
